# Role of AMP-activated protein kinase in cross-talk between apoptosis and autophagy in human colon cancer

**DOI:** 10.1038/cddis.2014.463

**Published:** 2014-10-30

**Authors:** X Song, S-Y Kim, L Zhang, D Tang, D L Bartlett, Y T Kwon, Y J Lee

**Affiliations:** 1 Department of Surgery, University of Pittsburgh, Hillman Cancer Center, Pittsburgh, PA 15213, USA; 2 Department of Pharmacology and Chemical Biology, School of Medicine, University of Pittsburgh, Pittsburgh, PA 15213, USA; 3 Protein Metabolism Medical Research Center and Department of Biomedical Science, College of Medicine, Seoul National University, Seoul 110-799, South Korea

## Abstract

Unresectable colorectal liver metastases remain a major unresolved issue and more effective novel regimens are urgently needed. While screening synergistic drug combinations for colon cancer therapy, we identified a novel multidrug treatment for colon cancer: chemotherapeutic agent melphalan in combination with proteasome inhibitor bortezomib and mTOR (mammalian target of rapamycin) inhibitor rapamycin. We investigated the mechanisms of synergistic antitumor efficacy during the multidrug treatment. All experiments were performed with highly metastatic human colon cancer CX-1 and HCT116 cells, and selected critical experiments were repeated with human colon cancer stem Tu-22 cells and mouse embryo fibroblast (MEF) cells. We used immunochemical techniques to investigate a cross-talk between apoptosis and autophagy during the multidrug treatment. We observed that melphalan triggered apoptosis, bortezomib induced apoptosis and autophagy, rapamycin caused autophagy and the combinatorial treatment-induced synergistic apoptosis, which was mediated through an increase in caspase activation. We also observed that mitochondrial dysfunction induced by the combination was linked with altered cellular metabolism, which induced adenosine monophosphate-activated protein kinase (AMPK) activation, resulting in Beclin-1 phosphorylated at Ser 93/96. Interestingly, Beclin-1 phosphorylated at Ser 93/96 is sufficient to induce Beclin-1 cleavage by caspase-8, which switches off autophagy to achieve the synergistic induction of apoptosis. Similar results were observed with the essential autophagy gene, autophagy-related protein 7, -deficient MEF cells. The multidrug treatment-induced Beclin-1 cleavage was abolished in Beclin-1 double-mutant (D133A/D146A) knock-in HCT116 cells, restoring the autophagy-promoting function of Beclin-1 and suppressing the apoptosis induced by the combination therapy. These observations identify a novel mechanism for AMPK-induced apoptosis through interplay between autophagy and apoptosis.

Colorectal cancer ranks third in major causes of cancer-related mortality worldwide, appearing in ~150 000 new cases in the United States annually, and ~20–50% of colorectal cancer patients display hepatic metastases.^1, 2^ Current standard therapies for treating metastatic colon cancer include chemotherapy and biological therapy followed by tumor resection, up front tumor resection followed by systemic therapy, radiofrequency ablation, thermal ablation, selective internal radiation therapy and hyperthermic isolated hepatic perfusion (IHP) therapy.^3, 4, 5, 6, 7^ Although these therapies are somewhat effective, more effective novel regimens are still needed to improve the survival of patients with liver metastases from colorectal cancer.

As unresectable liver metastases from colorectal cancer are difficult to treat by single modality, we have spent several years developing a multimodality approach for hyperthermic IHP therapy. We previously investigated the mechanism of the synergy between hyperthermia, biological agents (TNF-related apoptosis-inducing ligand/mapatumumab) and chemotherapeutic agent (oxaliplatin).^8, 9, 10^ However, the clinical grade of those biological agents is no longer available after newly merged companies decided not to produce them. We then investigated potential replacement drugs, which are already Food and Drug Administration (FDA) approved. We screened several FDA-approved drugs including melphalan, chlorquine, bortezomib, carbamazepine, celecoxib, cetuximab and rapamycin using cytoxicity assay. We found that MBR (melphalan+bortezomib+rapamycin) treatment has the best cytotoxic effect on colon cancer cells and also on colon cancer stem cells. Currently, 2807 clinical trials are listed for colon cancer; of these, 225 studies and 195 studies are related to FOLFOX (folinic acid+fluorouracil+oxaliplatin) therapy and FOLFIRI (folinic acid+fluorouracil+irinotecan) therapy, respectively. There are only seven studies for IHP and, specifically, there is no clinical trial with MBR for hyperthermic IHP therapy.

Apoptosis is a major cytotoxic mechanism of chemotherapy; stress-induced apoptosis often proceeds through the intrinsic pathway where permeabilization of the mitochondrial outer membrane releases cytochrome *c* and activates the caspase cascade.^11, 12^ Melphalan hydrochloride (trade name Alkeran), which is commonly used in IHP, leads to double-stranded DNA breaks and subsequent cell death through a caspase-mediated, apoptotic pathway.^13, 14^

Bortezomib, the first clinically available proteasome inhibitor, possesses antitumor activity in a variety of human cancers and is often used in the treatment of hematological malignancies. It can induce both proapoptotic effects, including the induction of Bik, Bim and Noxa proteins, and antiapoptotic effects, including the accumulation of Mcl-1 and HSP70, as well as autophagic formation.^15, 16, 17^

mTOR (mammalian target of rapamycin) is known to be well conserved and ubiquitously expressed in endothelial cells and is involved in cell energy metabolism, cell growth, apoptosis and autophagy. Several human cancers display mTOR hyperactivation, thus making mTOR an attractive target in cancer therapy.^18^ Sirolimus, known as rapamycin, an mTOR inhibitor, has relatively low cytotoxic activity. Better therapeutic outcomes should be obtained by using rapamycin in combination with other anticancer agents.^19, 20^ In this study, we observed that melphalan triggered apoptosis, bortezomib induced both apoptosis and autophagy, rapamycin caused autophagy and the combinatorial treatment promoted mitochondrial dysfunction and synergistic apoptosis.

Hallmarks of cancer cells include uncontrolled growth, evasion of apoptosis, immortality, ability to invade other tissues and altered cellular metabolism.^21^ In our study, we investigated the effect of multidrug treatment on cellular metabolism. This study revealed that the combinatorial treatment altered cellular metabolism and induced energy sensor AMP-activated protein kinase (AMPK) activation at two stages in the process, resulting in Beclin-1 phosphorylation and autophagy starting with the early stage and Beclin-1 cleavage by caspase-8 and apoptosis concurrent with the late stage. Our observations provided evidence that AMPK has an important role in cross-talk between autophagy and apoptosis.

## Results

### MBR synergistically induced cytotoxicity and apoptosis

**Figure 1:**
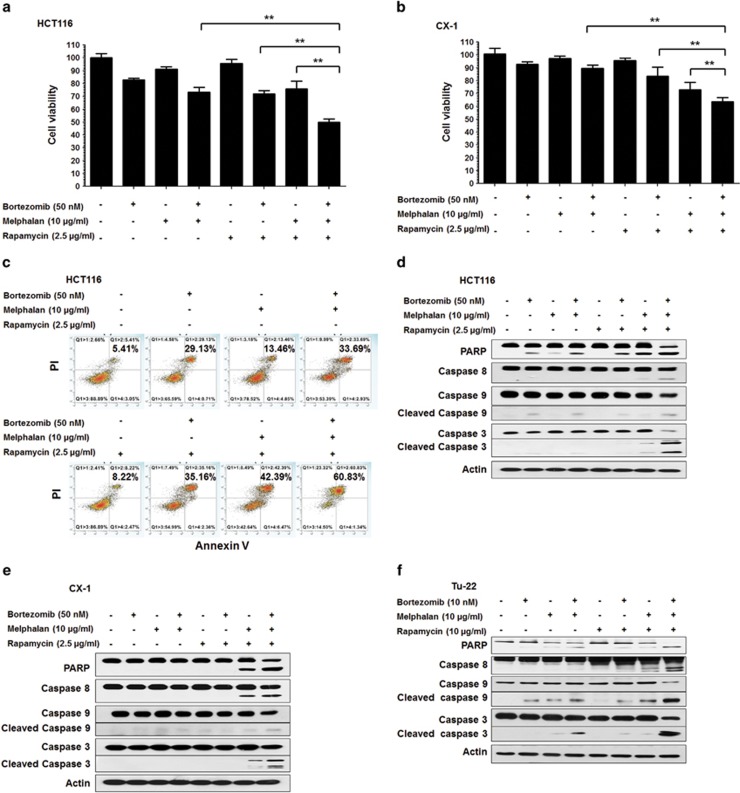
MBR induced cytotoxicity and apoptosis. (**a** and **b**) HCT116 (**a**) and CX-1 (**b**) cells were treated with MBR for 24 h. Cell viability was analyzed by MTS assay. Error bars represent S.D. from triplicate experiments. **P<0.01 represents a statistically significant difference. (**c**) HCT116 cells were treated with MBR for 24 h and cells were stained with fluorescein isothiocyanate (FITC)-Annexin V and PI. Apoptosis was detected by the flow cytometric assay. (**d–f**) After treatment, the cleavage of caspase-8, caspase-9, caspase-3 and PARP was detected by immunoblotting in HCT116 (**d**), CX-1 (**e**) and Tu-22 (**f**) cells. Actin was used to confirm the equal amount of proteins loaded in each lane

To investigate the effect on cell viability of the application of MBR, human colorectal carcinoma HCT116 and CX-1 cells were treated with a combination of 10 *μ*g/ml melphalan, along with 50 nM bortezomib and/or 2.5 *μ*g/ml rapamycin for 24 h. Cell viability was determined by 3-(4,5-dimethylthiazol-2-yl)-5-(3-carboxymethoxyphenyl)-2-(4-sulfophenyl)-2*H*-tetrazolium (MTS) assay. As shown in Figures 1a and b, synergistic effect was observed in MBR compared with any single treatment or bitreatment in both cell lines (*P*<0.01). Our observation was confirmed by combination index (CI) analysis; CI values were <1 (Table 1). In Figure 1c, apoptotic cells detected by Annexin V/PI assay were observed in the upper right quadrant of each plot. Our data clearly show that treatment with MBR enhanced synergistic induction of apoptotic death. These synergistic effects were due to an increased activation of caspases, and thus, the hallmark of apoptosis, poly (ADP-ribose) polymerase (PARP) cleavage (Figures 1d and e). Similar results were observed in human colon cancer stem Tu-22 cells (Figure 1f).

### MBR-induced mitochondrial dysfunction and AMPK activation

**Figure 2:**
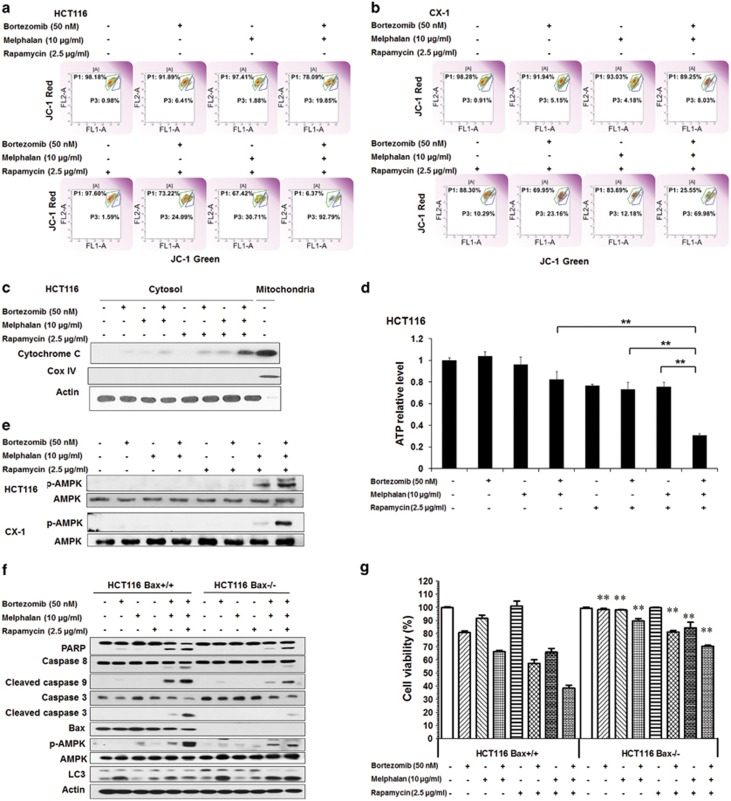
MBR-induced mitochondrial dysfunction and AMPK activation. (**a** and **b**) HCT116 (**a**) and CX-1 (**b**) cells were treated with MBR for 24 h, stained with JC-1 mitochondrial membrane potential detection kit and analyzed by flow cytometry. (**c**) After treatment, cytochrome *c* release into the cytosol was determined by immunoblotting for cytochrome *c* in the cytosolic fraction of HCT116 cells. Mitochondrial fraction was used as the control. Cox IV was used as a mitochondrial marker and actin as a cytosolic marker. (**d**) After treatment, ATP levels were assessed by using an ATP Assay Kit (Perkin-Elmer, Akron, OH, USA) in HCT116 cells. Data represent relative ATP levels, with control cells set as 100% (mean±S.D., *n*=3, **P<0.01). (**e**) After treatment, the activation of AMPK-*α* was detected by immunoblotting in HCT116 and CX-1 cells. (**f**) Parental HCT116 (HCT116 Bax^+/+^) and Bax-knockout HCT116 Bax^−/−^ cells were treated with MBR for 24 h and immunoblotted with anti-PARP, anti-caspase-8, anti-caspase-9, anti-caspase-3, anti-phospho-AMPK-*α*, anti-AMPK-*α* or anti-LC3 antibody. Actin was used to confirm the equal amount of proteins loaded in each lane. (**g**) HCT116 Bax^+/+^ and Bax^−/−^ cells were treated with MBR for 24 h. Cell viability was analyzed by MTS assay. Error bars represent S.D. from triplicate experiments. **P<0.01 represents a statistically significant difference

Stress-induced apoptosis often proceeds through the intrinsic pathway mediated by THE mitochondria. In Figures 2a and b, the upper left quadrant of each plot displays cells with intact mitochondrial membrane potential, whereas the lower right quadrant displays cells with impaired mitochondrial membrane potential. A significant shift occurred to the lower right part of the quadrants in the treatment with MBR. Figure 2c indicates that more cytochrome *c* was released during multidrug treatment. We then assessed the ATP production as the mitochondria are known to have an important role in providing overall cellular energy supply. ATP production was markedly decreased during the treatment with MBR in HCT116 cells (Figure 2d) and CX-1 cells (data not shown). Figure 2e shows that MBR induced a large amount of phosphorylation (activation) of AMPK. We then investigated whether Bcl-2-associated X protein (Bax) was involved in the combinatorial treatment-induced apoptosis. Data from Figure 2f clearly demonstrates that MBR-induced apoptosis and caspase activation were effectively suppressed in Bax-deficient cells, indicating that the synergy of MBR-associated apoptosis is partially mediated through Bax. Interestingly, AMPK activation was also decreased in HCT116 Bax^−/−^ cells, implying that Bax contributes to AMPK activation. The hallmark of autophagy, microtubule-associated protein 1A/1B-light chain 3 (LC3)-II, increased markedly in bortezomib-treated cells and mildly in rapamycin-treated cells but no significant increase with melphalan treatment. Notably, there was a significant decrease of LC3-II in the treatment of MBR compared with that of bortezomib alone. Interestingly, LC3-II was increased in the treatment of MBR in HCT116 Bax^−/−^ cells compared with that of HCT116 Bax^+/+^ cells, indicating that autophagy was increased in the apoptosis-suppressed cells (Figure 2f). These results indicate a cross-talk between autophagy and apoptosis during treatment with MBR. Figure 2g shows that cytotoxicity was significantly decreased in HCT116 Bax^−/−^ compared with HCT116 Bax^+/+^ (*P*<0.01).

### Interplay between autophagy and apoptosis during treatment with MBR

**Figure 3:**
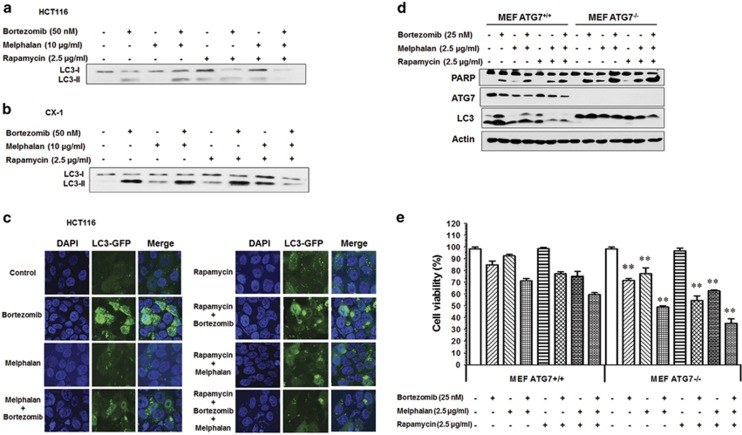
Interplay between autophagy and apoptosis during treatment with MBR. HCT116 (**a**) and CX-1 (**b**) cells were treated with MBR for 24 h, and LC3-I and LC3-II were detected by immunoblotting. (**c**) LC3 puncta formation was examined using confocal microscopy in HCT116 cells. (**d**) MEF ATG7^+/+^ and MEF ATG7^−/−^ cells were treated with MBR for 24 h, and PARP, ATG7 and LC3 were detected by immunoblotting. Actin was used to confirm the equal amount of proteins loaded in each lane. (**e**) MEF ATG7^+/+^ and MEF ATG7^−/−^ were treated with MBR for 24 h. Cell viability was analyzed by MTS assay. Error bars represent S.D. from triplicate experiments. **P<0.01 represents a statistically significant difference

To further investigate a relationship between apoptosis and autophagy during treatment with MBR, we examined autophagy using as an autophagy-specific marker both processing of LC3-I into LC3-II using immunoblot assay and also green fluorescent protein (GFP)-LC3 puncta formation using confocal microscopy. We observed that LC3-II (Figures 3a and b) and LC3 puncta formation (Figure 3c) increased significantly in bortezomib alone or in combination with melphalan or rapamycin. However, MBR treatment suppressed LC3-II or LC3 puncta formation compared with bortezomib alone. It is known that the classical autophagy pathway is dependent on Beclin-1, autophagy-related protein 7 (ATG7), and so on.^22^ Figure 3d shows that MBR treatment-induced apoptosis was enhanced in mouse embryo fibroblast (MEF) ATG7^−/−^ cells. Similar results were observed by MTS assay (Figure 3e). These results suggest that inhibition of autophagy enhances multidrug-induced apoptosis.

### The role of AMPK in MBR-induced apoptosis

**Figure 4:**
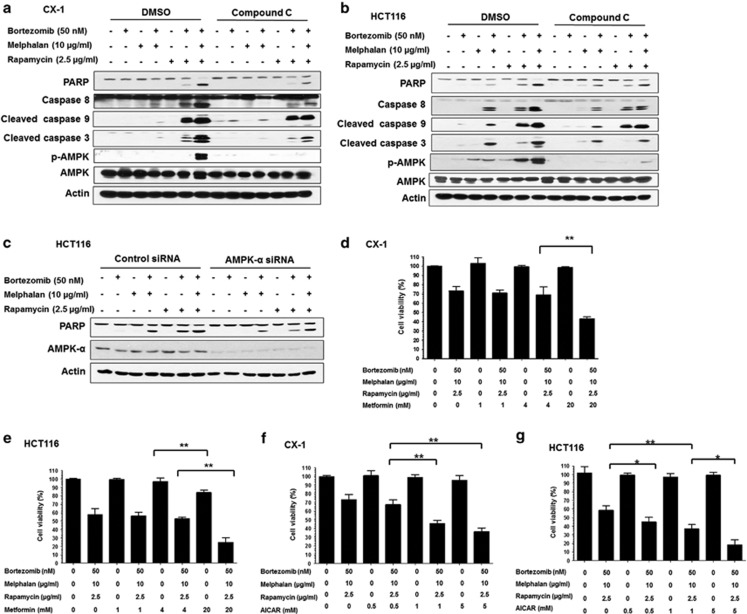
The role of AMPK in the MBR-induced apoptosis. (**a** and **b**) CX-1 (**a**) and HCT116 (**b**) cells were pretreated with 10 *μ*M AMPK inhibitor compound C followed by treatment with MBR for 24 h. After treatment, PARP, caspase-8, caspase-9, caspase-3, p-AMPK and AMPK were detected by immunoblotting. (**c**) HCT116 cells were transfected with nonsense sequence (control) or AMPK-*α* siRNA targeting AMPK-*α* mRNA. After 48 h, cells were treated with MBR. The levels of AMPK-*α* and PARP were detected by immunoblotting. Actin was used as a loading control. (**d** and **e**) CX-1 (**d**) and HCT116 (**e**) cells were pretreated for 30 min with metformin (1–20 mM) followed by treatment with MBR for 24 h. Cell viability was analyzed by MTS assay. Error bars represent S.D. from triplicate experiments. **P<0.01 represents a statistically significant difference. (**f** and **g**) CX-1 (**f**) and HCT116 (**g**) cells were pretreated for 30 min with AICAR (0.5–5 mM) followed by treatment with MBR for 24 h. Cell viability was analyzed by MTS assay. Error bars represent S.D. from triplicate experiments. *P<0.05 and **P<0.01 represents a statistically significant difference

To further investigate a cross-talk between autophagy and apoptosis, we hypothesized that enhancement of MBR-induced apoptosis is mediated through the activation of AMPK. To test this hypothesis, we added an AMPK inhibitor, compound C, in the absence or presence of MBR. Figures 4a and b show that MBR-induced apoptosis was partially suppressed by the addition of compound C, inhibiting the activation of AMPK-*α*. This observation was confirmed by knocking down the expression of AMPK-*α*. Figure 4c shows that a combination of AMPK-*α*1 and AMPK-*α*2 small interfering RNA (siRNA) downregulated AMPK-*α* expression level and partially suppressed MBR-induced apoptosis, which was consistent with the effects of compound C. Our results suggest that MBR-induced AMPK activation has an important role in the cross-talk between autophagy and apoptosis. To further investigate the role of AMPK activators metformin and AICAR (aminoimidazole carboxamide ribonucleotide) in MBR-induced cytotoxicity, cells were treated with metformin/AICAR in the absence or presence of MBR. As shown in Figure 4d, metformin alone did not affect CX-1 cell viability. However, MBR-induced cytotoxicity was significantly enhanced in the presence of 20 mM metformin. Similar results were observed in HCT116 cells (Figure 4e). Figures 4f and g show that AICAR alone did not affect cell viability in CX-1 and HCT116 cells but MBR-induced cytotoxicity was enhanced in the presence of AICAR.

### AMPK activation-induced Beclin-1 phosphorylation at Ser 93/96 and Beclin-1 cleavage

**Figure 5:**
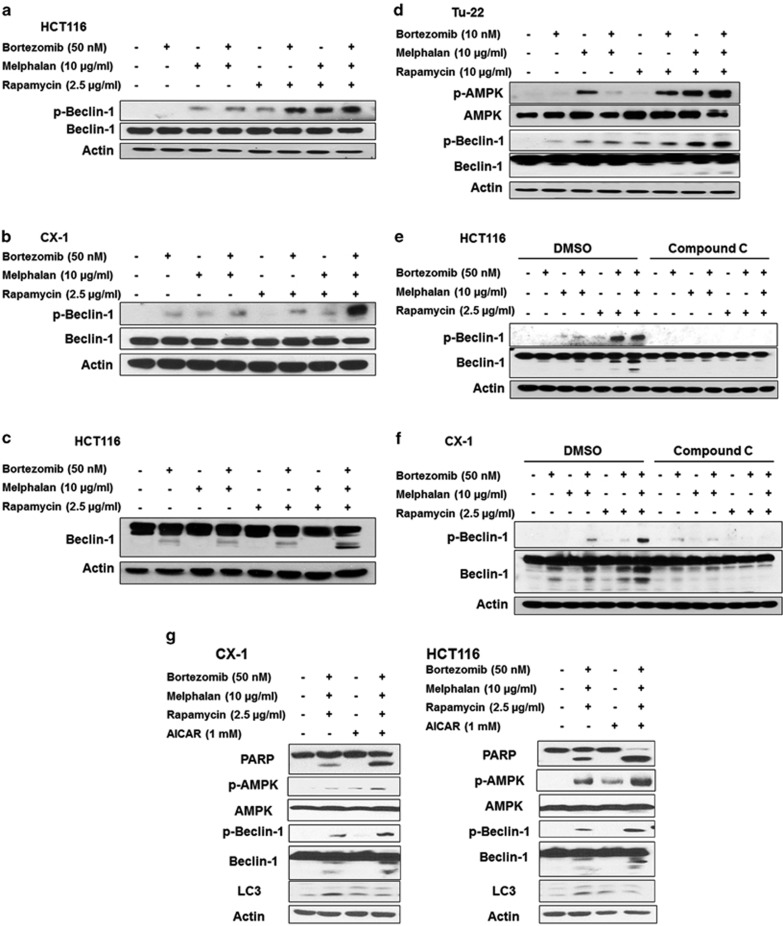
AMPK-*α* activation-induced Beclin-1 phosphorylation at Ser 93/96 and Beclin-1 cleavage. (**a** and **b**) HCT116 (**a**) and CX-1 (**b**) cells were treated with MBR for 24 h and p-Beclin-1 (Ser 93/96) and Beclin-1 were detected by immunoblotting in HCT116 and CX-1 cells. (**c**) After treatment, Beclin-1 cleavage was detected by immunoblotting in HCT116 cells. (**d**) Tu-22 cells were treated with MBR for 24 h and p-AMPK, AMPK, p-Beclin-1 (Ser 93/96) and Beclin-1 were detected by immunoblotting. (**e** and **f**) HCT116 (**e**) and CX-1 (**f**) cells were pretreated with 10 *μ*M compound C followed by MBR for 24 h, and Beclin-1 phosphorylation at Ser 93/96 and Beclin-1 cleavage were detected by immunoblotting. (**g**) CX-1 and HCT116 cells were pretreated for 30 min with 1 mM AICAR followed by treatment with MBR for 24 h. After treatment, PARP, p-AMPK, AMPK, p-Beclin-1 (Ser 93/96), Beclin-1 and LC3 were detected by immunoblotting. Actin was used to confirm the equal amount of proteins loaded in each lane

To examine the role of AMPK in the cross-talk between autophagy and apoptosis, we further investigated AMPK downstream molecules. Recently, AMPK was reported to phosphorylate Beclin-1 at Ser 91/94 for mouse or Ser 93/96 for human upon glucose starvation.^23^ C-terminal fragment of Beclin-1 localizes at the mitochondria, inducing cytochrome *c* release and thus enhancing the apoptosis.^24^ We observed Beclin-1 phosphorylation at Ser 93/96 in HCT116 and CX-1 cells (Figures 5a and b), as well as Beclin-1 cleavage in HCT116 cells (Figure 5c), during the treatment with MBR. Similar results were observed in Tu-22 cells (Figure 5d). We also observed that AMPK inhibitor, compound C, inhibited Beclin-1 phosphorylation at Ser 93/96 and Beclin-1 cleavage in both cell lines (Figures 5e and f). Figure 5g shows that 1 mM AICAR alone, which activated AMPK, did not induce apoptosis in CX-1 and HCT116 cells. However, AICAR enhanced MBR-induced apoptosis. We also observed that MBR-induced PARP cleavage, Beclin-1 phosphorylation at Ser 93/96 and Beclin-1 cleavage were enhanced in the presence of AICAR in both cell lines. AICAR alone increased the level of LC3-II (autophagy). However, a combinatorial treatment of MBR and AICAR reduced MBR-induced LC3-II. These results suggest a strong correlation between AMPK activation, Beclin-1 cleavage and switching the mode of cell death from autophagy to apoptosis.

### The kinetics of AMPK-*α* activation and Beclin-1 phosphorylation and cleavage in the treatment of MBR

**Figure 6:**
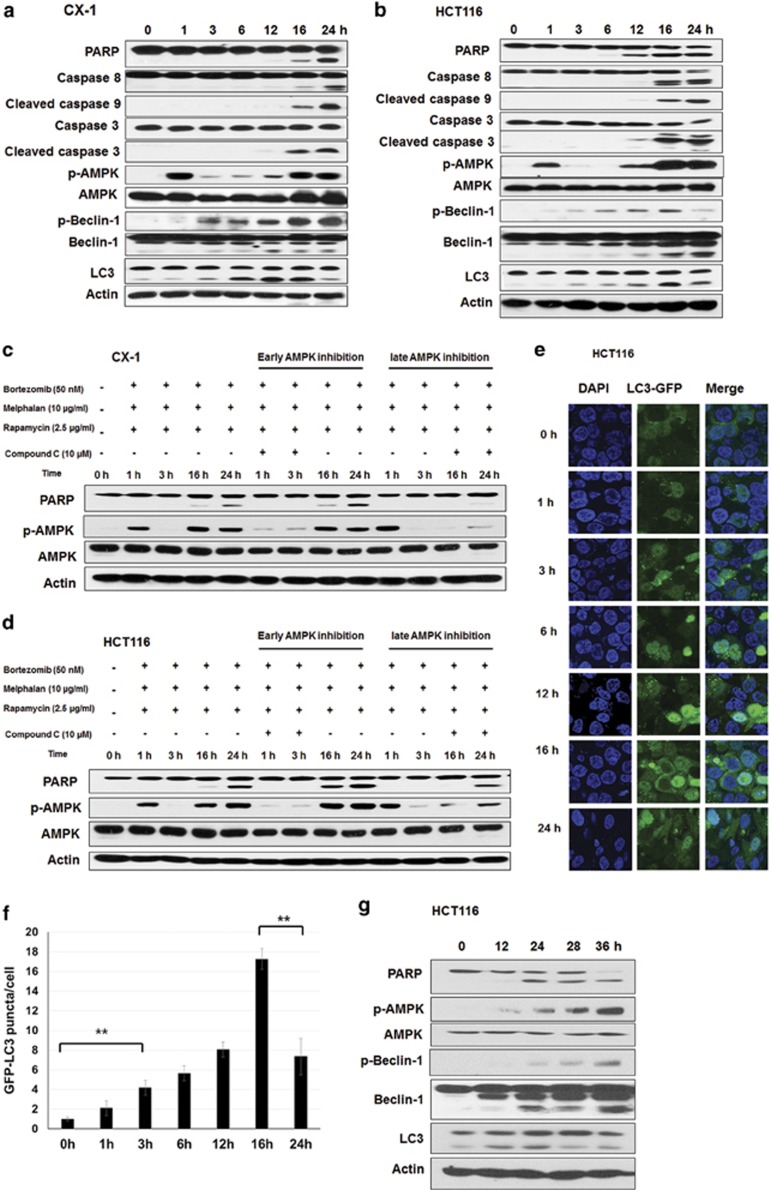
The kinetics of AMPK-*α* activation and Beclin-1 phosphorylation and cleavage during treatment with MBR. CX-1 (**a**) and HCT116 (**b**) cells were treated with MBR for various times (1–24 h). After treatment, cell lysates were immunoblotted with anti-PARP, anti-caspase-8, anti-caspase-9, anti-caspase-3, anti-phospho-AMPK-*α*, anti-AMPK-*α*, anti-phospho-Beclin-1, anti-Beclin-1 or anti-LC3 antibody. Actin was used to confirm the equal amount of proteins loaded in each lane. (**c** and **d**) CX-1 (**c**) and HCT116 (**d**) cells were treated with MBR in the presence of 10 *μ*M compound C from 0 to 4 h (at early time point and washed with phosphate-buffered saline at 4 h) or from 10 to 24 h (at late time point). After treatment, PARP, p-AMPK, AMPK and actin were detected by immunoblotting. (**e**) After treatment, LC3 punta formation was analyzed with confocal microscope in HCT116 cells. (**f**) Quantification of GFP-LC3 puncta formations. **P<0.01 represents a statistically significant difference. (**g**) HCT116 cells were treated with MBR for various times (12–36 h). After treatment, cell lysates were immunoblotted with anti-PARP, anti-phospho-AMPK-*α*, anti-AMPK-*α*, anti-phospho-Beclin-1, anti-Beclin-1 or anti-LC3 antibody. Actin was used to confirm the equal amount of proteins loaded in each lane

Next, we examined the kinetics of MBR-induced apoptosis. As shown in Figures 6a and b, MBR-induced apoptosis was increased as time progressed. In CX-1 cells, AMPK was phosphorylated (activated) very early at 1 h and then decreased. Notably, activation of AMPK was increased again at around 16 h in a sustained manner. Beclin-1 phosphorylation at Ser 93/96 was markedly increased after 3 h. Beclin-1 cleavage increased after 12 h when apoptosis occurred and then apoptosis was significantly increased. We also monitored autophagy in CX-1 cells after MBR treatment, using as an autophagy-specific marker processing of LC3-I into LC3-II. Interestingly, during the first 16 h, MBR treatment co-occurred with increased autophagy indicated by increased LC3-II levels. But then, reduced levels of LC3-II were observed at 24 h after treatment, implying that once caspases were fully activated, autophagy levels were diminished. Similar results were obtained in HCT116 cells, which were more sensitive towards the treatment of MBR compared with CX-1 cells (Figure 6b). To further test the differential role of AMPK activation at different stage, we used compound C to inhibit transiently AMPK at early stage (from 0 to 4 h and washed at 4 h with phosphate-buffered saline) and late stage (from 10 to 24 h) separately. As shown in Figures 6c and d, we observed that inhibiting AMPK at early time point enhanced MBR-induced apoptosis, whereas inhibiting AMPK at late time point abolished MBR-induced apoptosis in CX-1 and HCT116 cells.

We further examined the kinetics of autophagy during treatment with MBR; GFP-LC3 puncta formation was investigated. Figures 6e and f show that LC3 puncta formation was detected at 3 h, and increased until 16 h and then decreased ~2-fold at 24 h compared with that of 16 h of MBR treatment. Moreover, Figure 6g shows that PARP cleavage, AMPK activation and Beclin-1 phosphorylation at Ser 93/96, as well as Beclin-1 cleavage, consistently increased even at 36 h. We also observed that LC3-II was markedly decreased at 28 and 36 h. These data suggest that early AMPK activation-induced Beclin-1 phosphorylation and resulted in autophagy, whereas sustained late AMPK activation co-occurring with the activation of caspases induced Beclin-1 cleavage, enhancing apoptotic cell death.

### Beclin-1 phosphorylation at Ser 91/94 is sufficient to induce Beclin-1 cleavage and thus contributes to the synergistic induction of apoptosis

**Figure 7:**
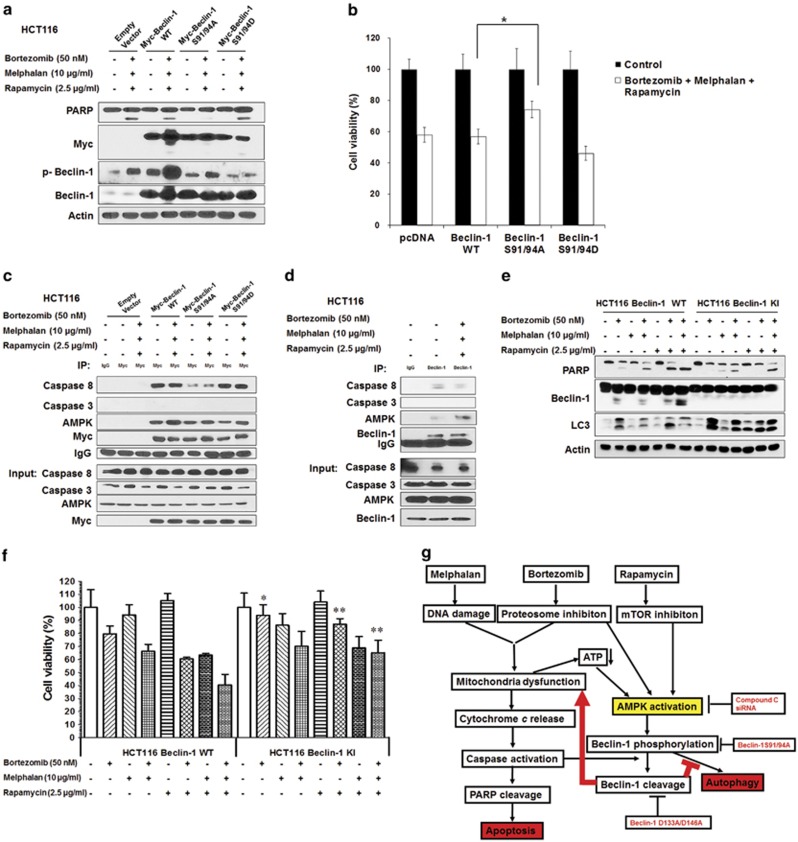
Beclin-1 phosphorylation at Ser 91/94 was a prerequisite for Beclin-1 cleavage and the cleavage of Beclin-1 contributed to the synergistic induction of apoptosis. HCT116 cells were transiently transfected with empty vector (pcDNA), Myc-Beclin-1 WT, Myc-Beclin-1 S91/94A and Myc-Beclin-1 S91/94D; 48 h later cells were treated with MBR for 24 h. (**a**) PARP, Myc, phosphorylated Beclin-1 and Beclin-1 were detected by immunoblotting. Actin was shown as an internal standard. (**b**) Cell viability was determined by MTS assay. *P<0.05 represents a statistically significant difference. (**c**) Cell lysates were immunoprecipitated with anti-Myc antibody or IgG and then immunoblotted with anti-caspase-8/3, anti-AMPK-*α* or anti-Myc antibody (upper panels). The presence of caspase-8/3, AMPK-*α* and Myc in the lysates was examined (lower panels). (**d**) HCT116 cells were treated with MBR for 24 h and cell lysates were immunoprecipitated with anti-Beclin-1 antibody or IgG and then immunoblotted with anti-caspase-8/3, anti-AMPK-*α* or anti-Beclin-1 antibody (upper panels). The presence of caspase-8/3, AMPK-*α* and Beclin-1 in the lysates was examined (lower panels). (**e**) Parental HCT116 (HCT116 Beclin-1 WT) and HCT116 Beclin-1 KI (DM; D133A/D146A) cells were treated with MBR and immunoblotted with anti-PARP, anti-Beclin-1 or anti-LC3 antibody. Actin was used to confirm the equal amount of proteins. (**f**) After treatment, cell viability was determined by MTS assay. *P<0.05 and **P<0.01 represent a statistically significant difference. (**g**) A schematic diagram of the role of AMPK in cross-talk between apoptosis and autophagy

To evaluate the effect of Beclin-1 phosphorylation at Ser 91/94 on its cleavage, we transiently transfected HCT116 cells with empty vector (pcDNA), wild-type murine Beclin-1, murine Beclin-1 S91/94A (residues 91 and 94 serine were replaced by alanine: dominant-negative mutant) and murine Beclin-1 S91/94D (residues 91 and 94 serine were replaced by aspartic acid: dominant-positive mutant) and then treated with MBR for 24 h. As shown in Figure 7a, phosphorylation of wild-type murine Beclin-1, but not Beclin-1 S91/94A or Beclin-1 S91/94D, was elevated by treatment with MBR. Interestingly, Beclin-1 S91/94A reduced MBR-induced PARP cleavage and cell death (Figures 7a and b), indicating that Beclin-1 phosphorylation at Ser 91/94 is a prerequisite for Beclin-1 cleavage.

Cleavage of Beclin-1 occurred with caspase activation. As caspase activation occurs during apoptotic signaling, we investigated if Beclin-1 and caspases bind with each other and thus execute their function during the treatment. Interestingly, as shown in Figure 7c, Beclin-1 and its mutant types all bound with caspase-8, whereas binding with caspase-3 was hardly detected. Of note, the binding affinity of caspase-8 with Beclin-1 S91/94A was weaker than that of Beclin-1 WT and Beclin-1 S91/94D, which needs further study. We also observed that the binding affinity of AMPK with Beclin-1 was increased after treatment of MBR in both Beclin-1 WT and its mutant types. These observations were confirmed by immunoprecipitating assay with endogenous Beclin-1 proteins (Figure 7d). Data from Figure 7d reveal that endogenous Beclin-1 indeed associated with AMPK and caspase-8 but not caspase-3. Notably, the binding affinity of Beclin-1 and AMPK was increased after MBR treatment.

D133 and D146 of Beclin-1 are known to be cleaved by caspase-8 during apoptosis. HCT116 Beclin-1 knock-in (Beclin-1 KI) cell line with double-mutant (DM; D133A/D146A) was generated by homologous recombination.^25^ As shown in Figures 7e and f, MBR-induced Beclin-1 cleavage was abolished in Beclin-1 KI cells, restoring Beclin-1's autophagy-promoting function as well as suppressing MBR-induced apoptosis.

Taken together, we summarized our observations in Figure 7g. MBR treatment causes mitochondrial dysfunction and AMPK activation. AMPK-mediated Beclin-1 phosphorylation is sufficient to induce Beclin-1 cleavage and thus contributes to the synergistic induction of apoptosis by decreasing autophagy; specifically, blocking Beclin-1 cleavage promotes autophagy and suppresses the apoptosis induced by MBR.

## Discussion

Multidrug treatment can potentially overcome the resistance developed by single agents as well as reduce their side effects.^26, 27^ Here we have presented a novel combination treatment of melphalan in combination with the proteasome inhibitor bortezomib and the mTOR inhibitor rapamycin for colon cancer cells, as well as colon cancer stem cells, and demonstrated that this multidrug treatment-induced synergistic apoptosis mediated by AMPK activation through facilitation of Beclin-1 cleavage.

In recent research, the relationship between cancer cell growth and cell metabolism has been emphasized, highlighting the role of AMPK as one of the main players.^28^ AMPK is a eukaryotic heterotrimeric (*α*, *β* and *γ*) protein kinase activated in response to environmental stresses such as glucose deprivation and hypoxia, which produce changes in cellular ATP levels, resulting in phosphorylation of AMPK at Thr 172. Chemotherapy with certain drugs can activate AMPK to increase cell death and apoptosis.^29, 30^ Rapamycin and UPS (ubiquitin–proteasome system) inhibitors may upregulate AMPK activity.^31, 32^ It has been found that AMPK is involved in both cell survival and cell death pathways, depending on the severity of energy stresses, drug action and cell types. Many researchers have reported that activation of AMPK induces apoptosis in cancer cells by inhibition of fatty acid synthase, phosphorylation of p53 or stimulation of the mitochondrial apoptotic pathway.^33, 34, 35, 36, 37, 38^

In our current study, we observed significant AMPK activation in MBR treatment. AMPK inhibitor compound C and AMPK-*α* siRNA suppressed MBR-induced apoptosis, indicating the proapoptotic effect of AMPK in the combinatorial treatment of MBR. This observation was also supported by experiments using AMPK activator metformin and AICAR. Interestingly, we observed that AMPK was activated at two stages. Early AMPK activation-induced autophagy, whereas late AMPK activation resulted in significant apoptosis. Autophagy, a catabolic degradation process, has recently emerged as an important stress response and cell death regulatory mechanism. The process of conventional macroautophagy is dependent on Atg7 (ubiquitin-activating enzyme (E1)-like) and Beclin-1.^39^ We observed that MBR treatment-induced apoptosis was enhanced in MEF ATG7^−/−^ cells, indicating that blockage of autophagy promoted apoptosis in MBR treatment. We also observed that autophagy decreased in the combination of MBR compared with bortezomib alone. Thus, these results indicate the role of AMPK in cross-talk between apoptosis and autophagy in the multidrug treatment.

Beclin-1, a mammalian homolog of yeast Atg6, functions in autophagy by initiating autophagosome formation in combination with Vps34 (a class III PI-3 kinase that generates PtdIns3P) and with Vps15 and Atg14;^40^ Beclin-1 then regulates autophagosome maturation by binding to UVRAG and Rubicon.^41^ Cross-talk between apoptosis and autophagy was associated with caspase-mediated cleavage of Beclin-1, which both destroys its proautophagic activity^22, 25, 42, 43^ and can then amplify mitrochondrion-mediated apoptosis through the cleaved C-terminal fragment.^24^ However, how Beclin-1 cleavage is regulated is largely unknown.

Beclin-1 phosphorylation sites reported include: Thr 119,^44^ Ser 14,^45^ Ser 93/96^23^ and Ser 234/295.^46^ Recently, AMPK was reported to phosphorylate directly Beclin-1 at Ser 93/96 for activating proautophagy Vps34 complex and subsequently inducing autophagy.^23^ The question remains as to how AMPK-mediated phosphorylation of Beclin-1 at Ser 93/96 regulates caspase-8-associated cleavage of Beclin-1. Here, we reported that Beclin-1 phosphorylation at Ser 93/96 is a prerequisite for Beclin-1 cleavage and thus contributes to the synergistic induction of apoptosis. AMPK inhibitor compound C inhibited both Beclin-1 phosphorylation at Ser 93/96 and Beclin-1 cleavage. In addition, Myc-tagged murine Beclin-1 S91/94A mutant was resistant to cleavage and suppressed MBR-induced apoptosis. Moreover, the binding affinity of Beclin-1 S91/94A with caspase-8 was decreased, providing one possible mechanism of AMPK-mediated Beclin-1 cleavage through its phosphorylation and formation of an AMPK–Beclin-1–caspase-8 complex. Beclin-1 DM (D133A/D146A) knock-in HCT116 cells partially abolished the apoptosis, confirming the role of Beclin-1 cleavage in the multidrug treatment.

Taken together, we present here that a novel multidrug treatment of chemotherapeutic agent in combination with proteasome inhibitor and mTOR inhibitor induced robust apoptosis in colon cancer cells as well as colon cancer stem cells, an apoptotic process that is linked with altered cellular metabolism and AMPK activation. We believe that these results demonstrate for the first time that the induction of apoptosis by AMPK is associated with Beclin-1 cleavage through Beclin-1 phosphorylation at Ser 93/96. Melphalan, bortezomib and rapamycin are all commonly used FDA-approved drugs and could be considered for colorectal hepatic metastases treatment in clinics.

## Materials and Methods

### Cell cultures and transfection

Human colorectal carcinoma CX-1 cells, which were obtained from Dr. JM Jessup (National Institutes of Health, Bethesda, MD, USA),^47^ were cultured in RPMI-1640 medium (Gibco-BRL, Grand Island, NY, USA) containing 10% fetal bovine serum (HyClone, Logan, UT, USA). The human colorectal carcinoma HCT116 Bax-containing (Bax^+/+^) and Bax-deficient (Bax^−/−^) cell lines were kindly provided by Dr. B Vogelstein (Johns Hopkins University, Baltimore, MD, USA); these cell lines were cultured in McCoy's 5A medium (Gibco-BRL) containing 10% fetal bovine serum.^48^ Human colon cancer stem Tu-22 cells were established by Dr. E Lagasse (University of Pittsburgh, Pittsburgh, PA, USA) and cultured in DMEM/F12 medium (Gibco-BRL) containing 0.5% fetal bovine serum (HyClone) and 1% insulin, transferrin and selenium (Fisher Scientific, Pittsburgh, PA, USA).^49^ MEF ATG7^+/+^ and MEF ATG7^−/−^ cell lines, a kind gift from Dr. Masaaki Komatsu (The Tokyo Metropolitan Institute of Medical Science, Tokyo, Japan), were cultured in DMEM medium (Gibco-BRL) containing 10% fetal bovine serum. All the cells were kept in a 37 °C humidified incubator with 5% CO_2_. For transient transfection, cells were transfected with Lipofectamine 2000 (Life Technologies, Carlsbad, CA, USA) and were treated 48 h after transfection.

### Reagents and antibodies

Bortezomib and melphalan were from Millennium Pharmaceuticals (Cambridge, MA, USA) and GlaxoSmithKline (Coraopolis, PA, USA), respectively. Rapamycin, metformin, aminoimidazole carboxamide ribonucleotide and protease inhibitor cocktail were obtained from Sigma-Aldrich (St. Louis, MO, USA). AMPK inhibitor (compound C) was from Calbiochem (Darmstadt, Germany). Myc-Beclin-1 WT, Myc-Beclin-1 S91/94A and Myc-Beclin-1 S91/94D were kindly provided by Dr. Kun-Liang Guan (University of California at San Diego, La Jolla, CA, USA). Anti-Myc, anti-Bax, anti-caspase-8, anti-caspase-9, anti-caspase-3, anti-phosphorylated AMPK-*α*/AMPK-*α*, anti-phosphorylated Beclin-1 (Ser 93/96), anti-COX-IV and anti-PARP antibody were from Cell Signaling (Danvers, MA, USA). Anti-LC3 and anti-actin antibody were from Sigma-Aldrich. Anti-Beclin-1 and anti-cytochrome *c* antibody were from BD PharMingen (San Jose, CA, USA).

### MTS assays

MTS studies were carried out using the Promega CellTiter 96 AQueous One Solution Cell Proliferation Assay (Promega, Madison, WI, USA). CX-1 cells (1 × 10^5^) were grown in RPMI-1640 medium containing 10% fetal bovine serum in tissue culture-coated 96-well plates and treated with drugs for 24 h. Cells were then treated with 20 *μ*l MTS/phenazine methosulfate solution for 1–4 h at 37 °C. Absorbance at 490 nm was determined using an enzyme-linked immunosorbent assay plate reader.

### CI analysis

CIs were calculated using CompuSyn software program (ComboSyn Inc., Paramus, NJ, USA). Base on CI values, extent of synergism/antagonism is determined. In general, CI value below 1 suggests synergy, whereas CI value above 1 indicates antagonism between the drugs. CI values in the range of 0.9–1.10 would mainly indicate additive effects, those between 0.9 and 0.85 would suggest slight synergy, those in the range of 0.7–0.3 are indicative of moderate synergy and those <0.3 would suggest strong synergy.

### Annexin V binding

Cells were treated with drugs, harvested by trypsinization, washed with serum-free medium and suspended in binding buffer (Annexin V-fluorescein isothiocyanate (FITC) Staining Kit; BD PharMingen). This cell suspension was stained with mouse anti-human Annexin V antibody and propidium iodide (PI) and immediately analyzed by flow cytometry.

### Immunoprecipitation

Briefly, cells were lysed in CHAPS lysis buffer with protease inhibitor cocktail (Calbiochem). The lysate (0.5**–**1 mg) was incubated with 1.5 *μ*g of anti-Myc/Beclin-1 antibody or rabbit/mouse IgG (Santa Cruz, Dallas, TX, USA) at 4 °C overnight, followed by the addition of protein G PLUS-agarose beads (Santa Cruz) and rotation at room temperature for 2 h followed by immunoblot analysis.

### Immunoblot analysis

Cells were lysed with Laemmli lysis buffer and boiled for 10 min. The protein content was measured with BCA Protein Assay Reagent (Pierce, Rockford, IL, USA), separated by sodium dodecyl sulfate-polyacrylamide gel electrophoresis (SDS-PAGE) and electrophoretically transferred to nitrocellulose membrane. The nitrocellulose membrane was blocked with 5% nonfat dry milk in PBS-Tween-20 (0.1%, v/v) for 1 h and incubated with primary antibody at room temperature for 2 h. Horseradish peroxidase-conjugated anti-rabbit or anti-mouse IgG was used as the secondary antibody. Immunoreactive protein was visualized by the chemiluminescence protocol.

### JC-1 mitochondrial membrane potential assay

After drug treatment, cells were stained with JC-1 Mitochondrial Membrane Potential Detection Kit (Invitrogen, Carlsbad, CA, USA) for 10 min and analyzed by flow cytometry. Fluorescence intensity was measured with the FACScan flow cytometer (Beckman Coulter, Hialeah, FL, USA). Results were analyzed with CellQuest software (Becton Dickinson Immunocytometry Systems, San Jose, CA, USA).

### ATP assay

The ATP content in whole-cell extracts was determined with a luminescent ATP Detection Kit (ATPlite, Perkin-Elmer, Akron, OH, USA) according to the manufacturer's instructions. The luminescence intensity was measured by using a microplate reader (Synergy 2; BioTek Instruments, Winooski, VT, USA). In parallel, the cell numbers in whole-cell samples were counted by Trypan blue exclusion assay. The results were expressed as relative ATP level compared with controls after normalizing for cell numbers.

### Measurement of cytochrome *c* release

To determine the release of cytochrome *c* from the mitochondria, cells growing in 100 mm dishes were used. After drug treatment, mitochondrial and cytosolic fractions were prepared using Mitochondrial Fractionation Kit (Active Motif, Carlsbad, CA, USA) following company instructions with reagents included in the kit. Cytosolic fractions were subjected to SDS-PAGE gel electrophoresis and analyzed by immunoblotting using anti-cytochrome *c* antibody.

### Knockdown of AMPK-*α* with siRNA oligomers

To generate AMPK-*α* knockdown cells, cells were transfected with 10 nM of siRNA AMPK-*α*1 and AMPK-*α*2 and control siRNA from Sigma-Aldrich, using Lipofectamine 2000. Target sequences for preparing siRNAs of human AMPK-*α*1 and *α*2 were as follows: 5'-AGUGAAGGUUGGCAAACAUTT-3' (sense strand) and 5'-AUGUUUGCCAACCUUCACUTT-3' (complement strand) for human AMPK-*α*1; 5'-GGAAGGUAGUGAAUGCAUATT-3' (sense strand) and 5'- UAUGCAUUCACUACCUUCCTT-3' (complement strand) for human AMPK-*α*2.^50^ Expression levels were determined by immunoblot analysis.

### Analysis of GFP-LC3 puncta formation

Stably transduced HCT116 tumor cells expressing the GFP-LC3 gene were generated by lentiviral transfection of the pCT-autophagosome-GFP Vector (Lentiviral Laboratory of the Vector Core Facility of the University of Pittsburgh) and selected with puromycin. HCT116 cells were treated with 10 *μ*g/ml melphalan, along with 50 nM bortezomib and/or 2.5 *μ*g/ml rapamycin for 24 h. Cells were washed three times with PBS, followed by fixation in 4% paraformaldehyde for 15 min. Nuclei were stained with DAPI (Cell Signaling). Slides were mounted and visualized in 0.4 *μ*m sections using an OlympusFluoview 1000 confocal microscope and the companion software FV10-ASW2.1 (Olympus, Center Valley, PA, USA) under a × 63 oil immersion objective. GFP-LC3 puncta formation was quantified by counting at least 300 cells for each sample and plotted as mean±S.D. of three independent experiments.

### Statistical analysis

Statistical analysis was carried out using Graphpad InStat 3 software (GraphPad Software, San Diego, CA, USA). Data showing comparisons between two groups were assessed using the Student's *t*-test. Comparisons among more than two groups were carried out using ANOVA with the appropriate *post hoc* testing. Statistical significance is marked with asterisks (*P<0.05 and **P<0.01).

## Figures and Tables

**Table 1:** Combination index for melphalan in combination with bortezomib and rapamycin^a^

**Combination therapy**			**Combination index**	
**Melphalan (*μ*g/ml)**	**Bortezomib (nM)**	**Rapamycin (*μ*g/ml)**	**HCT116**	**CX-1**
10	50	2.5	0.33	0.52
20	100	5	0.35	0.80
40	200	10	0.49	0.95

a Calculated by Compusyn software for HCT116 and CX-1 colon cancer cells

## Abbreviations

AICAR: aminoimidazole carboxamide ribonucleotide
AMPK: 5' adenosine monophosphate-activated protein kinase
ATP: adenosine-5'-triphosphate
ATG: autophagy-related protein
BAX: Bcl-2-associated X protein
FDA: Food and Drug Administration
FITC: fluorescein isothiocyanate
IHP: isolated hepatic perfusion
LC3: microtubule-associated protein 1A/1B-light chain 3
mTOR: mammalian target of rapamycin
PARP: poly (ADP-ribose) polymerase
PAGE: polyacrylamide gel electrophoresis
SDS: sodium dodecyl sulfate
TRAIL: TNF-related apoptosis-inducing ligand
